# Supplementary material for: Serological detection of Mycobacterium Tuberculosis complex infection in multiple hosts by One Universal ELISA
Source: PLoS One. 2021 Oct 7;16(10):e0257920. doi: 10.1371/journal.pone.0257920 (PMC8496862; doi:10.1371/journal.pone.0257920)
Supplement: S1 Table — (DOCX) [file pone.0257920.s001.docx]

**S1 Table Information on the cattle confirmed positive by mycobacterial culture and PCR and serological testing with ELISA**

| **Number** | **Samples used for bacterial culture** | **Gross pathological change** | **Bacterial culture^1^** | **Tissue direct PCR typing ^2^** | **MMEC/AG-iELISA** | **IDEXX kit** |
| --- | --- | --- | --- | --- | --- | --- |
| **1** | Mediastinal lymph nodes | Yes | *M. bovis* | *M. bovis* | Negative | Negative |
| **2** | Lung | Yes | *M. bovis* | *M. bovis* | Negative | Negative |
| **3** | Spleen | Yes | *M. bovis* | *M. bovis* | Positive | Positive |
| **4** | Hilar lymph nodes | Yes | *M. bovis* | *M. bovis* | Positive | Positive |
| **5** | Hilar lymph nodes | Yes | *M. bovis* | *M. bovis* | Negative | Negative |
| **6** | Mesenteric lymph nodes | Yes | *M. bovis* | *M. bovis* | Negative | Negative |
| **7** | Liver | Yes | *M. bovis* | *M. bovis* | Positive | Positive |
| **8** | Hilar lymph nodes | Yes | *M. bovis* | *M. bovis* | Positive | Positive |
| **9** | Hilar lymph nodes | Yes | *M. bovis* | Negative | Positive | Negative |
| **10** | Mesenteric lymph nodes | Yes | *M. bovis* | *M. bovis* | Positive | Positive |
| **11** | Hilar lymph nodes | Yes | *M. bovis* | *M. bovis* | Negative | Negative |
| **12** | Mesenteric lymph nodes | Yes | *M. bovis* | *M. bovis* | Positive | Negative |
| **13** | Spleen | Yes | *M. bovis* | Negative | Positive | Positive |
| **14** | Mesenteric lymph nodes | Yes | *M. bovis* | Negative | Positive | Negative |
| **15** | Lung | Yes | *M. bovis* | *M. bovis* | Negative | Negative |
| **16** | Lung | Yes | *M. bovis* | *M. bovis* | Negative | Negative |
| **17** | Lung | Yes | *M. bovis* | *M. bovis* | Positive | Positive |

^1:^ Template used for PCR typing was the bacterial genome extracted from cultured bacteria.

^2:^ Template used for PCR typing was the bacterial genome extracted from tissue with pathological change.
