# Supplementary material for: Serological detection of Mycobacterium Tuberculosis complex infection in multiple hosts by One Universal ELISA
Source: PLoS One. 2021 Oct 7;16(10):e0257920. doi: 10.1371/journal.pone.0257920 (PMC8496862; doi:10.1371/journal.pone.0257920)
Supplement: S2 Table — (DOCX) [file pone.0257920.s002.docx]

**S2 Table Determination of MMEC and AG-HRP dilutions**

| **Concentrations of**  **AG-HRP (μg/mL）** |  | **Concentrations of MMEC (ng/mL）** | | | | |
| --- | --- | --- | --- | --- | --- | --- |
|  |  | **200.00** | **100.00** | **50.00** | **25.00** | **12.50** |
| **4.00** | P^1^ | 3.9806 | 3.1909 | 1.8603 | 1.0611 | 0.5649 |
|  | N^2^ | 0.2106 | 0.1236 | 0.1474 | 0.1508 | 0.0930 |
|  | P/N | 18.9012 | 25.8163 | 12.6208 | 7.0365 | 6.0742 |
| **1.00** | P | 3.5480 | 2.4408 | 1.3191 | 0.7298 | 0.4021 |
|  | N | 0.1435 | 0.1104 | 0.1020 | 0.1034 | 0.0693 |
|  | P/N | 24.7247 | 22.1087 | 12.9323 | 7.0580 | 5.8023 |
| **0.40** | P | 3.0972 | 1.9278 | 1.2587 | 0.5836 | 0.3214 |
|  | N | 0.1095 | 0.0791 | 0.0912 | 0.0840 | 0.0634 |
|  | P/N | 28.2849 | 24.3717 | 13.8015 | 6.9476 | 5.0694 |
| **0.27** | P | 2.8425 | 1.7812 | 0.8581 | 0.5210 | 0.2966 |
|  | N | 0.1135 | 0.0882 | 0.0934 | 0.0758 | 0.0606 |
|  | P/N | 25.0441 | 20.1950 | 9.1874 | 6.8734 | 4.8944 |

^1^ P: The values of OD of the positive control serum.

^2^ N: The values of OD of the negative control serum.
