# Supplementary material for: Serological detection of Mycobacterium Tuberculosis complex infection in multiple hosts by One Universal ELISA
Source: PLoS One. 2021 Oct 7;16(10):e0257920. doi: 10.1371/journal.pone.0257920 (PMC8496862; doi:10.1371/journal.pone.0257920)
Supplement: S3 Table — (DOCX) [file pone.0257920.s003.docx]

**S3 Table Determination of serum dilution**

|  | **Dilutions** | | | | | | | |
| --- | --- | --- | --- | --- | --- | --- | --- | --- |
|  | **10** | **25** | **50** | **100** | **200** | **400** | **800** | **1600** |
| **P^1^** | 3.126 | 2.615 | 1.93 | 1.113 | 0.799 | 0.432 | 0.221 | 0.101 |
| **N^2^** | 0.161 | 0.116 | 0.089 | 0.066 | 0.058 | 0.061 | 0.052 | 0.033 |
| **P/N** | 19.4161 | 22.5431 | 21.6854 | 16.8636 | 13.7759 | 7.0820 | 4.2500 | 1.9057 |

^1^ P: The values of OD of the positive control serum.

^2^ N: The values of OD of the negative control serum.
