# Supplementary material for: Serological detection of Mycobacterium Tuberculosis complex infection in multiple hosts by One Universal ELISA
Source: PLoS One. 2021 Oct 7;16(10):e0257920. doi: 10.1371/journal.pone.0257920 (PMC8496862; doi:10.1371/journal.pone.0257920)
Supplement: S5 Table — (DOCX) [file pone.0257920.s005.docx]

**S5 Table Analytical sensitivity of MMEC/AG-iELISA and IDEXX kit in serological diagnosis of bovine TB caused by *Mycobacterium bovis***

|  | **S/P values** | | | | | |
| --- | --- | --- | --- | --- | --- | --- |
| **Dilutions** | **1.6 × 2^12^** | **1.6 × 2^13^** | **1.6 × 2^14^** | **1.6 × 2^15^** | **1.6 × 2^16^** | **1.6 × 2^17^** |
| **MMEC/AG-iELISA** | 0.905 | 0.670 | 0.463 | 0.259 | 0.145 | 0.058 |
| **IDEXX kit** | 4.082 | 2.180 | 0.918 | 0.241 | 0.039 | -0.035 |

Note: The cut-off value of both MMEC/AG-iELISA and IDEXX kit was S/P = 0.3.
