# Supplementary material for: Serological detection of Mycobacterium Tuberculosis complex infection in multiple hosts by One Universal ELISA
Source: PLoS One. 2021 Oct 7;16(10):e0257920. doi: 10.1371/journal.pone.0257920 (PMC8496862; doi:10.1371/journal.pone.0257920)
Supplement: S6 Table — (DOCX) [file pone.0257920.s006.docx]

**S6 Table Comparison of MMEC/AG-iELISA and IDEXX kit in serological detection of bTB**

|  | | **IDEXX kit** | | |
| --- | --- | --- | --- | --- |
|  |  | Positive | Negative | Total |
| **MMEC/AG-iELISA** | Positive | 120 | 0 | 120 |
|  | Negative | 0 | 242 | 242 |
|  | Total | 120 | 242 | 362 |
