# Supplementary material for: Serological detection of Mycobacterium Tuberculosis complex infection in multiple hosts by One Universal ELISA
Source: PLoS One. 2021 Oct 7;16(10):e0257920. doi: 10.1371/journal.pone.0257920 (PMC8496862; doi:10.1371/journal.pone.0257920)
Supplement: S8 Table — (DOCX) [file pone.0257920.s008.docx]

**S8 Table** **Comparison of MMEC/AG-iELISA and INGEZIM kit in the serological detection of sheep TB**

| **Test method** | **No. test positive/total**  **positive** | **No. test negative/total**  **negative** | **Sensitivity**  **(95%** **CI: down, upper)** | **Specificity**  **(95% CI: down, upper)** |
| --- | --- | --- | --- | --- |
| **MMEC/AG-iELISA** | 46/46 | 113/113 | 100.00%  (95.00%, 100.00%) | 100.00%  (96.80%, 100.00%) |
| **INGEZIM kit** | 14/46 | 113/113 | 30.43%  (17.70%, 45.80%) | 100.00%  (96.80%, 100.00%) |
