# Supplementary material for: Serological detection of Mycobacterium Tuberculosis complex infection in multiple hosts by One Universal ELISA
Source: PLoS One. 2021 Oct 7;16(10):e0257920. doi: 10.1371/journal.pone.0257920 (PMC8496862; doi:10.1371/journal.pone.0257920)
Supplement: S9 Table — (DOCX) [file pone.0257920.s009.docx]

**S9 Table Analytical sensitivity of MMEC/AG-iELISA and INGEZIM kit in detection of sheep TB caused by *Mycobacterium bovis***

|  | **S/P or OD values** | | | | | | | | |
| --- | --- | --- | --- | --- | --- | --- | --- | --- | --- |
| **Dilutions** | **50** | **100** | **200** | **400** | **800** | **1600** | **3200** | **6400** | **12800** |
| **MMEC/AG-iELISA** | 1.444 | 1.249 | 1.085 | 0.958 | 0.669 | 0.474 | 0.259 | 0.132 | 0.066 |
| **INGEZIM kit** | 1.801 | 0.928 | 0.483 | 0.250 | 0.154 | 0.134 | 0.076 | 0.067 | 0.061 |

Note: The cut-off value of MMEC/AG-iELISA was 0.22 (S/P), while that of INGEZIM kit was 0.39 (OD).
