# Supplementary material for: Serological detection of Mycobacterium Tuberculosis complex infection in multiple hosts by One Universal ELISA
Source: PLoS One. 2021 Oct 7;16(10):e0257920. doi: 10.1371/journal.pone.0257920 (PMC8496862; doi:10.1371/journal.pone.0257920)
Supplement: S10 Table — (DOCX) [file pone.0257920.s010.docx]

**S10 Table Comparative tests with intradermal comparative test, MMEC/AG-iELISA, and PPD-ELISA to differentiate MTBC and NTM infected sheep**

| **Number** | **The increase in skin-fold thickness (mm)** | | **MMEC/AG-iELISA** | **PPD-ELISA** |
| --- | --- | --- | --- | --- |
|  | **Avain PPD injection site** | **Bovine PPD injection site** |  |  |
| **1** | 6.89 | 1.73 | Negative | Negative |
| **2** | 7.28 | 3.6 | Negative | Negative |
| **3** | 14.13 | 2.53 | Negative | Negative |
| **4** | 9.59 | 7.19 | Negative | Negative |
| **5** | 4.36 | 2.18 | Negative | Negative |
| **6** | 13.32 | 1.66 | Negative | Negative |
| **7** | 13.09 | 3.56 | Negative | Negative |
| **8** | 7.41 | 1.7 | Negative | Negative |
| **9** | 10.66 | 2.04 | Negative | Negative |
| **10** | 13.25 | 6.25 | Negative | Negative |
| **11** | 13.1 | 7.17 | Negative | Negative |
| **12** | 10.1 | 3.03 | Negative | Negative |
| **13** | 4.95 | 2.92 | Negative | Negative |
| **14** | 5.09 | 3.7 | Negative | Negative |
| **15** | 11.38 | 1.8 | Negative | Negative |
| **16** | 11.89 | 0.43 | Negative | Negative |
| **17** | 19.37 | 5.45 | Negative | Negative |
| **18** | 11.14 | 3.1 | Negative | Negative |
| **19** | 12.48 | 2.61 | Negative | Negative |
| **20** | 9.17 | 3.75 | Negative | Negative |
| **21** | 27.27 | 8.12 | Negative | Negative |
| **22** | 4.12 | 0.07 | Negative | Negative |
| **23** | 9.67 | 1.68 | Negative | Negative |
| **24** | 4.93 | 2.65 | Negative | Negative |
| **25** | 4.52 | 1.65 | Negative | Negative |
| **26** | 5.92 | 2.62 | Negative | Negative |
