# Supplementary material for: Serological detection of Mycobacterium Tuberculosis complex infection in multiple hosts by One Universal ELISA
Source: PLoS One. 2021 Oct 7;16(10):e0257920. doi: 10.1371/journal.pone.0257920 (PMC8496862; doi:10.1371/journal.pone.0257920)
Supplement: S12 Table — (DOCX) [file pone.0257920.s012.docx]

**S12 Table Analytical sensitivity of MMEC/AG-iELISA and INGEZIM kit in the detection of sika deer TB caused by *Mycobacterium bovis***

|  | **S/P or OD values** | | | | | | | |
| --- | --- | --- | --- | --- | --- | --- | --- | --- |
| **Dilutions** | **50** | **100** | **200** | **400** | **800** | **1600** | **3200** | **6400** |
| **MMEC/AG-iELISA** | 1.574 | 1.649 | 1.507 | 1.269 | 1.009 | 0.742 | 0.472 | 0.304 |
| **INGEZIM kit** | 3.269 | 3.118 | 2.873 | 1.913 | 0.976 | 0.462 | 0.273 | 0.153 |

Note: The cut-off value of MMEC/AG-iELISA was 0.54 (S/P), while that of INGEZIM kit was 0.39 (OD).
