# Supplementary material for: Serological detection of Mycobacterium Tuberculosis complex infection in multiple hosts by One Universal ELISA
Source: PLoS One. 2021 Oct 7;16(10):e0257920. doi: 10.1371/journal.pone.0257920 (PMC8496862; doi:10.1371/journal.pone.0257920)
Supplement: S13 Table — (DOCX) [file pone.0257920.s013.docx]

**S13 Table Analytical sensitivity of MMEC/AG-iELISA and INGEZIM kit in the diagnosis of roe deer TB caused by *Mycobacterium caprae***

|  | **S/P or OD450 values** | | | | | | |
| --- | --- | --- | --- | --- | --- | --- | --- |
| **Dilutions** | **10** | **25** | **50** | **100** | **200** | **400** | **800** |
| **MMEC/AG-iELISA** | 1.342 | 1.061 | 0.763 | 0.533 | 0.248 | 0.187 | 0.093 |
| **INGEZIM kit** | 0.101 | 0.135 | 0.080 | 0.077 | 0.085 | 0.107 | 0.041 |

Note: The cut-off value of MMEC/AG-iELISA was 0.54 (S/P), while that of INGEZIM kit was 0.39 (OD).
