# Supplementary material for: Serological detection of Mycobacterium Tuberculosis complex infection in multiple hosts by One Universal ELISA
Source: PLoS One. 2021 Oct 7;16(10):e0257920. doi: 10.1371/journal.pone.0257920 (PMC8496862; doi:10.1371/journal.pone.0257920)
Supplement: S15 Table — (DOCX) [file pone.0257920.s015.docx]

**S15 Table Analytical sensitivity of MMEC/AG-iELISA and INGEZIM kit in the detection of macaque monkey TB caused by *Mycobacterium bovis***

|  | **S/P or OD values** | | | | | | |
| --- | --- | --- | --- | --- | --- | --- | --- |
| **Dilutions** | **25** | **50** | **100** | **200** | **400** | **800** | **1600** |
| **MMEC/AG-iELISA** | 1.347 | 0.937 | 0.753 | 0.461 | 0.252 | 0.100 | 0.023 |
| **INGEZIM kit** | 0.071 | 0.086 | 0.053 | 0.045 | 0.051 | 0.06 | 0.042 |

Note: The cut-off value of MMEC/AG-iELISA was 0.17 (S/P), while that of INGEZIM kit was 0.39 (OD).
