# Supplementary material for: Serological detection of Mycobacterium Tuberculosis complex infection in multiple hosts by One Universal ELISA
Source: PLoS One. 2021 Oct 7;16(10):e0257920. doi: 10.1371/journal.pone.0257920 (PMC8496862; doi:10.1371/journal.pone.0257920)
Supplement: S1 Raw images — (PDF) [file pone.0257920.s017.pdf]

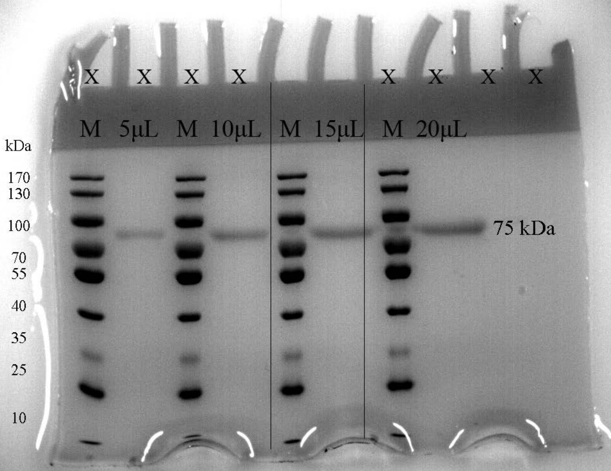


The coated fusion antigen MMEC was checked by 10% SDS-PAGE with different loading amount of 5µL, 10µL, 15µL, and 20µL

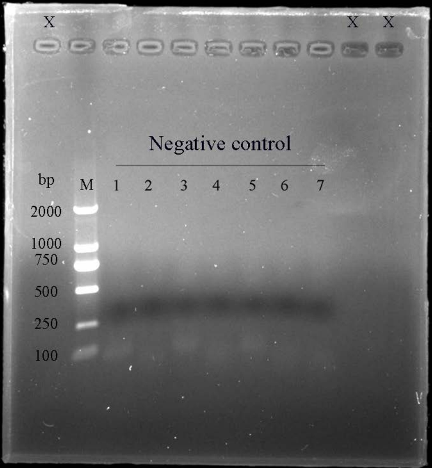


Multiplex PCR typing of the mycobacterium isolates from deer species.
Negative control was deionized water. Lanes: 1, 16S Rrna (543 bp); 2, Rv0577 (786 bp); 3, IS1561' (943 bp); 4, Rv1510 (1033 bp); 5, Rv1970 (1116 bp); 6, Rv3877/8 (999 bp); 7, Rv3120 (404 bp); M, 2000 bp DNA ladders.

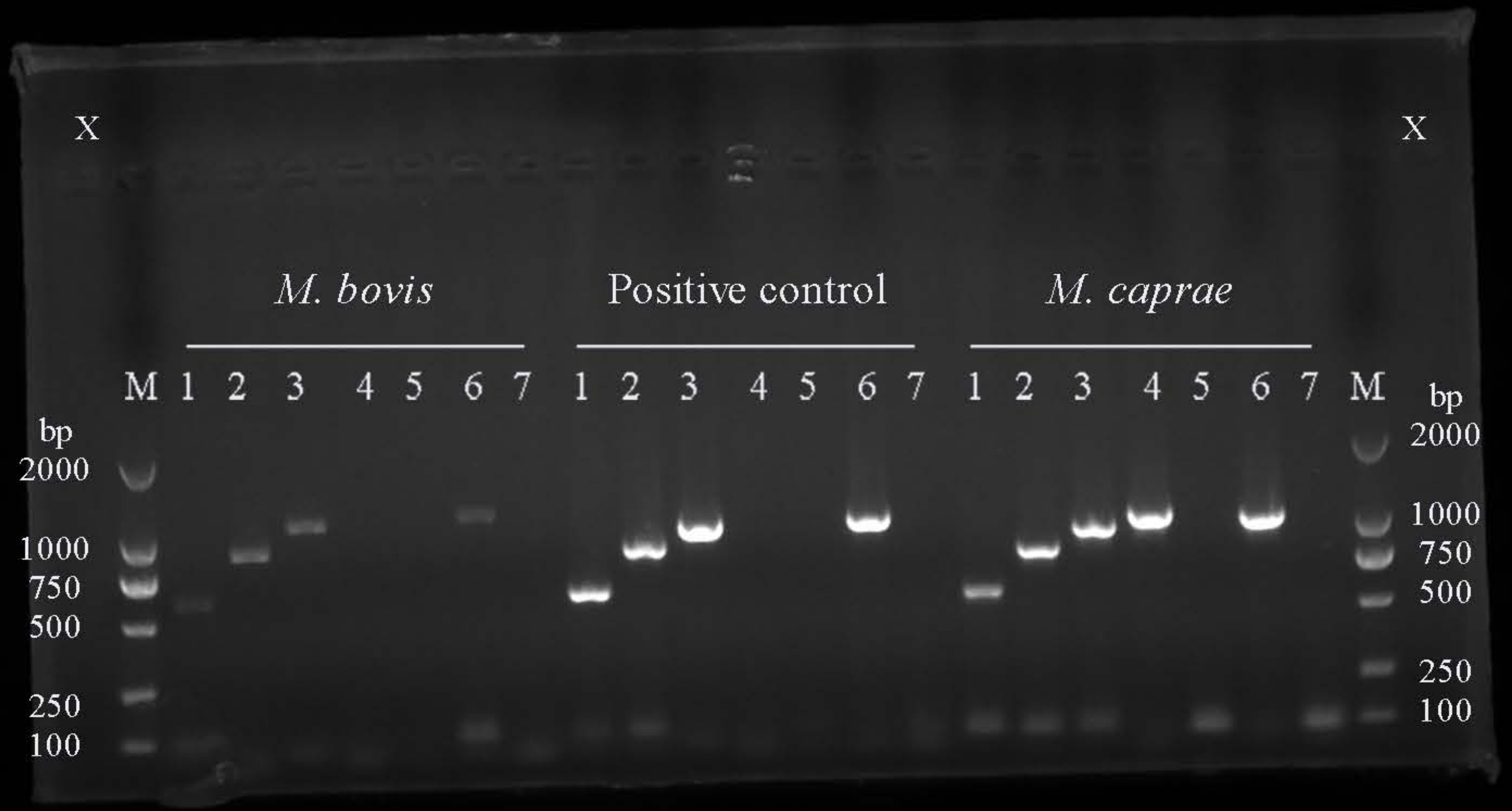


Multiplex PCR typing of the mycobacterium isolates from deer species. *M. bovis* was isolated from the tuberculous nodule in the lung of a Pere David's deer; Positive control was *M. bovis* ATCC19210; *M. caprae* was isolated from the abscesses in the right submaxillary face of a roe deer. Lanes: 1, 16S Rrna (543 bp); 2, Rv0577 (786 bp); 3, IS1561' (943 bp); 4, Rv1510 (1033 bp); 5, Rv1970 (1116 bp); 6, Rv3877/8 (999 bp); 7, Rv3120 (404 bp); M, 2000 bp DNA ladders.
