# Supplementary material for: Serological detection of Mycobacterium Tuberculosis complex infection in multiple hosts by One Universal ELISA
Source: PLoS One. 2021 Oct 7;16(10):e0257920. doi: 10.1371/journal.pone.0257920 (PMC8496862; doi:10.1371/journal.pone.0257920)
Supplement: Attachment — Submitted filename: Response to Reviewers.docx [file pone.0257920.s018.docx]

Response to Reviewer 1 Comments

Reviewer #1: This paper describes the development of a serological assay for species of the Mycobacterium tuberculosis complex or MTB is a variety of host species. I think this work is highly relevant as accurate, easy-to-use, diagnostics for animal TB are sorely lacking but pivotal to aid the control of TB. It needs to be noted however that serology/humoral immunity in TB is not very well understood and generally serological assays are not to be used as stand-alone assays as they show highly variable test performance. This assay shows promise, but it needs to be validated in a larger set of samples to access the true test performance as there may be some selection bias in the samples used in the present study. Furthermore, for some species there were rather few samples, so this needs to be addressed as well. That being said, I think the study has relevance, was well executed and should definitely be published. Please see my comments below.

Author response: We greatly appreciate for the positive comments.

General comments:

1. I couldn't find the all the data from the study. The authors declare it is all in the figures/tables or supplemental info, but I don't see for instance the outcomes of testing the sera at various dilutions. I think this should also be made available, to strengthen the final choice of serum dilution used in the assay.

Response 1: Thank you very much for this suggestion and very sorry to cause this confusion due to lack of the detailed data. As required, in the revised submission, the additional data were added as the supplementary tables (S4 Table for bovines, S7 for sheep, S11 for cervids, S14 for monkeys, and S16 for humans).

2. Adding to that, why was it chosen to only assay 2 serum dilutions (cattle) or 3 serum dilutions (in other species) rather than a range of serum dilutions in a checkerboard titration as is standard when developing a novel assay?

Response 2: Thank you very much for this comment. We are very sorry to have neglected the related background about the serum dilution and completely agree with you that a range of serum dilutions should be used in checkerboard titration to determine the optimal serum dilution. We actually followed this standard in this study. To determined optimum serum dilution, we performed checkerboard titration by using a range of serum dilutions with the positive bovine serum (the pooled 120 positive bovine sera) and negative bovine serum (the pooled 242 negative bovine sera) since the amount of these bovine sera could meet demands of repetitive tests. As a result, the optimal dilution was determined to be 1:100 (the addition table named as S3 table was provided in the revised version). Considering the difference of serum titers among various species, in practical tests for each species, the two or three dilutions are selected around the 1:100. Then sensitivity and specificity were compared and the dilution with the highest sensitivity and specificity was finally determined for each species. We added the ROC curves for other serum dilutions in the tests of each species to better show the evidence for decision-making (please see Fig.2, 3, 5, and 6).

3. The strength of the assay is that it is able to detect Ab to various members of MTBC in a number of species. However, in some cases it may be important to know with which mycobacterium the animal is infected (perhaps this is mostly true for humans, given that patients would receive different treatment for *M. tb* than *M. bovis*. Any comments on this? Also, have you considered testing serum of an animal (experimentally) infected with non-tuberculous mycobacteria (NTM) to determine the specificity of this assay further? Gcebe et al. 2016 found that ESAT6, CFP10 and MPB70 were also present in the genome/proteome of some NTM, so this could mean that animals infected with those species could test positive on this assay.

Response 3: Thank you very much for this smart comment. We agree with you that rapid identification of specific members of MTBC is desired to provide proper treatment or control measures in time for humans or animals. But it is quite difficult currently due to several factors including the chronic process of TB, a high proportion of persistent infection, the slow growth of MTBC, low efficiency to isolate the bacteria, and biosafety requirement for MTBC, etc. However, to quickly know the infected status would be very helpful to take further actions. To target this, we developed this MMEC/AG-iELISA.

We indeed considered the situation that animals were infected by NTM infection because ESAT6, CFP10, and MPB70 were also present in the genome/proteome of some NTM as you mentioned. Rather than experimentally infected the animals with NTM since it is difficult to get seroconversion animals by experimental infection due to the chronic progress characteristics of mycobacterial infection, we selected 23 sheep which were considered as NTM infection by comparative intradermal skin test since the increase of skin-fold thickness to the avian PPD was greater than bovine PPD and greater than 4mm, tested their serum samples with both MMEC/AG-iELISA and PPD-ELISA, and demonstrated all the serum samples were negative. These additional data were provided in S10 Table in the revised version.

4. In the abstract, you state this assay will help controlling TB. I feel like this is too strong. Animals are not likely to be culled on the basis of this assay's outcome alone. There is much more validation and evaluation needed before this happens. So I think this language needs to be a little less strong. It is definitely a good step in the right direction. Maybe use "surveillance" or "monitoring" instead of "controlling" or say that it is a step towards that.

Response 4: Thank you very much for this suggestion. As suggested, this sentence has been rewritten as follows: This method has a promising application in serological TB surveillance for multiple animal species thereby providing evidence for taking further action in TB control.

5. In the introduction (lines 65-67): This is not really true for animal TB. As I mentioned, humoral immunity is not that well understood and so serological assays are not used that much at all (even if a lot of research has focused on the development of these assays, mostly because the relative ease which with they can be used compared to IGRAs or the skin test). Furthermore, currently (besides humans) none of the species studied in this work are currently being vaccinated by *M. bovis* BCG (nor any other vaccine) because of the fact that animals vaccinated with BCG test positive on the traditional TB diagnostics.

Response 5: Thank you very much for this suggestion. We basically agree with your points.

Compared to the IGRAs or the skin test, the sensitivity of antibody tests for humoral immunity is lower. But as you mentioned, it is easy to be conducted. Furthermore, currently IGRAs and the skin test are only well-calibrated in bovines and humans. For many species of animals, serological testing is the most feasible way to systemically understand the background of MTBC infection.

Besides, we agree with you that domestic animals are not allowed to be vaccinated with BCG because of the lack of DIVA, BCG vaccination was performed in the badgers in the UK. Since the IGRAs DIVA based on RD derived antigens such as ESAT6/CFP10 have been generated to differentiate BCG vaccination and natural infection. As the great burden caused by the slaughtering of positive animals in developing countries, it would be possible to vaccinate domestic animals with BCG in the future.

In addition, by following your suggestion, this sentence has been rewritten as follows: Antibody detection is an ideal choice to identify the risk of TB transmission in multiple species due to its advantage of simple, easy, rapid, and high-throughput screening.

6. In section 2.2, it is stated that the human TB patients were infected with *M. tb*, was this confirmed using molecular techniques? Also, in the section on sheep, in the skin test, I was wondering whether the presence of clinical signs of a delayed-type hypersensitivity reaction were also noted as a positive skin test? Furthermore, it is mentioned that the sheep were subcutaneously injected twice with PPD-B to raise an antibody response. It is not mentioned where on the animal this was done? And was it done using a McLintock gun like used in skin testing or a syringe?

Response 6: Thank you very much for this comment. We are very sorry to have neglected the related details about the background of serum production. In the revised version, we added the related information as follows:

(1) Yes, the human TB patients were confirmed by the professional hospital followed the standard procedures including molecular techniques described previously (ref 26). To make it clear, the following description was added in the revised text. “Briefly, the 24 human serum samples from 24 TB patients were collected previously by the Tuberculosis department, Wuhan Medical Treatment Center and stored at -80℃ by this laboratory [26]. The TB patients were firstly suggested by clinical signs and chest X-ray test, and then confirmed by *M. tb* isolation from sputum with mycobacterial culture and molecular typing. The hospital used Xpert to rapidly type *M. tb* and test drug resistance gene *rpoB*, while we used multiplex PCR to further differentiate MTBC members described as follows. In addition, the 233 human negative sera were collected from healthy volunteers who were negative in intradermal tuberculin test (induration area < 5 mm), chest X-ray test, and *in vitro* blood IFN-γ release test.

(2) For the sheep TB skin test with the intradermal comparative test, we followed the OIE standard protocol for bovine TB test which states that the reaction was considered to be negative if the increase in skin-fold thickness at the bovine injection site was less than or equal to the increase in the skin-fold reaction at the avian injection site, and without clinical signs, such as diffuse or extensive oedema, exudation, necrosis, pain or inflammation of the lymphatic ducts in that region or the lymph nodes. The only clinical signs observed were skin redness and swelling at the avian PPD injection site in 26 sheep which were defined as NTM infection (S10 Table).

(3) For immunization with PPD-B, sheep were subcutaneously inoculated with 2500 IU/0.1 mL PPD-B in the center of the middle third of the neck with a 1 mL sterile syringe. This sentence has been rewritten as follows: the TB negative sheep were subcutaneously inoculated with 2500 IU/0.1 mL PPD-B and boosted with the same dose 30 days later in the center of the middle third of the neck with a 1 mL sterile syringe.

7. In the same section, concerning different animal species: Is it not a bit too confident to say that this assay can also be used for *M. caprae* on the basis of one serum from a confirmed *M. caprae* animal? Also, how was the causative agent in monkeys determined (in other words, was it definitely *M. bovis* and not *M. tb*?). Same for sika deer, was this confirmed *M. bovis* by molecular techniques?

Response 7: Thank you again for this critical comment. As you understand, identification of specific members of MTBC is quite difficult currently in different species for TB due to several factors including the chronic process of TB, a high proportion of persistent infection, the slow growth of MTBC, low efficiency to isolate the bacteria, and biosafety requirement for MTBC, etc.

(1) Regarding *M. caprae* infection, it is the first and only case we have confirmed with mycobacterial culture and molecular typing so far. Meanwhile, we tested the serum samples from the other three roe deer of the same group but without clinical signs and found they are positive too. Based on the sample number, we agree we should limit the extension of our finding. Therefore, we added the results of the other three serum samples from the animals of the same group and decreased confidence in the description by adding “likely” in the revised version.

Figure The other three roe deer cohabited with the *M. caprae* infected roe deer

(2) Regarding the monkey and sika deer tests, the MTBC infection status was determined by the upper eyelid intradermal PPD-B test, PPD-ELISA, and MPB83 sandwich ELISA for monkeys, while PPD-ELISA and MPB83 sandwich ELISA for sika deer. Since PPD-B causes cross-reaction between *M. tb* and *M. bovis* infection, the causative agent in monkeys and deer can’t be identified as *M. bovis* or *M. tb* except for the positive Pere David's deer with *M. bovis* infection and roe deer with *M. caprae* infection which were confirmed by mycobacterial culture and molecular technique (multiplex PCR) respectively by using the tissue samples or abscess samples.

(3) Since we couldn’t get more serum samples from the animals with a clear background of MTBC member infection, to further determine if our MMEC/AG-iELISA can detect *M. tb* infection, we used the serum samples from human TB patients whose causative agent was confirmed to *M. tb*. The results showed that MMEC/AG-iELISA could detect *M. tb* infection very well.

8. In section 2.5, it is explained that the optimal concentration for the MMEC and AG-HRP if the positive OD was close to 1 and the negative OD >10. This may be a naive question, but is this a standard approach? If not, why was this chosen as optimal? I am only aware of the method that looks at the plateauing of the OD values in checkerboard titrations.

Response 8: Thank you again for this smart comment. We are sorry to misinterpret the criteria. Yes, we indeed looked at the plateauing of the OD ratio of positive to negative (P/N), meanwhile considered the linear range of OD values and usage of the materials as less as possible to decrease production cost. Therefore, the optimal conditions were determined at the highest P/N value and/or the OD value of the positive serum was slightly over 1. We added the additional table and reference for this process (S3 Table Determination of serum dilution and reference [30]).

9. In section 2.6, for the ODnc and ODpc very specific numbers are given (0.09 and 1.26, respectively) but instead of using "=", the curly equal sign is used, which means "equals approximately". This is odd. Also if these were used as validation criteria (more appropriate term than "prerequisite"), what if the OD was not exactly that, did you disregard the results? Or did you use a range? Or was it valid as long as ODnc was smaller or equal to 0.09 and ODpc larger or equal to 1.26? If so, please use the appropriate symbols.

Response 9: Thank you very much for this important and kind comment. These specific numbers of OD_NC_ and OD_PC_ corresponded to the condition that the P/N value was the highest and the OD value of the positive serum was slightly over 1 when the optimal coated MMEC concentration and AG-HRP dilution were determined in checkerboard titration (S2 Table). We changed the expression in the revised version as suggested: Only when the average OD_NC_ was smaller or equal to 0.09, while the average OD_PC_ was larger or equal to 1.26 which were determined by the checkerboard titration, the test is valid.

10. In figure 1, the product looks much larger than 75kDa?

Response 10: Thank you very much for this careful comment. The recombinant plasmid was previously constructed in our lab, encoding the fusion protein MMEC which includes *mpb70*, *mpb83*, *cfp10*, and *esat6* gene, and the linker GSGGGGSGGGGSGS between each two genes. We confirmed the plasmid to be correct by digestion with *EcoRI* and *HindⅢ* and analysis on the coding sequence of MMEC by sequencing. Therefore, we think it might be the concentration of SDS-PAGE causing this difference. Then we re-ran the 10% SDS-PAGE and got the expected size of the band.

11. In section 3.2, sera from animals that were culture-confirmed *M. bovis* positive are tested on the two ELISAs, but the outcomes are called prevalences. This is not the appropriate term. It is the proportion of animals testing positive in a known cohort of *M. bovis* positive animals. A prevalence is when you test an entire herd/population for a disease and then report how may animals tested positive.

Response 11: Thank you very much for this correction. "prevalences" has been changed to "sensitivity" in the revised version.

12. In S1 Table, animals 9, 13 and 14 are *M. bovis* positive on bacterial culture, but not confirmed by PCR. How was bacterial culture assessed? Based on morphology, ZN staining? What growth medium was used? What PCR (gene target) was used? For 2/3 of these animals the MMEC/Ag-HRP was positive, but the IDEXX negative, for the other animals both ELISAs are positive. It is motioned that this attests to higher Se of the MMEC assay, but could it not also be a false-positive? Or do you suspect the PCR was false-negative? Please explain.

Response 12: Thank you very much for this comment. We are very sorry to have neglected the related details of the background about S1 Table.

The whole process that all the 17 cattle were diagnosed as *M. bovis* infection by mycobacterial culture and multiplex PCR typing as follows: the tissue with pathological change of the 17 cattle were collected in an abattoir. The collected samples were homogenized and decontaminated with 4% sodium hydroxide. After neutralization and centrifugation, the sediment is inoculated on 7H11 medium for a minimum of 8 weeks at 37°C with CO_2_ for MTBC culture. When colony growth is visible, the genome of the colony was extracted by boiling. Then, the subspecies of the MTBC were identified by multiplex PCR targeting *16S Rrna*, Rv0577, IS*1561’*, Rv1510, Rv1970, Rv3877/8, and Rv3120 gene as described before [27]. Briefly, each PCR mixture was prepared with 25 μL of PCR Master, 20 μL of water, 2.5 μL of dimethyl sulfoxide, 1 μL of each primer at 20 μM, and 0.5 μL of DNA. PCR amplifications were performed with an initial denaturation step of 5 min at 94°C followed by 35 cycles of 60 s at 94°C, 60 s at 60°C, and 90 s at 72°C, and ending with a final elongation step for 10 min at 72°C. PCR products were visualized by 1% agarose gel electrophoresis. The PCR products were sent to be sequenced for each strain and confirmed to be correct. As a result, all the 17 cattle were determined to be infected by *M. bovis*.

Meanwhile, the genome of MTBC in collected tissue was also extracted directly with nucleic acid extraction instruments, the subspecies of the MTBC were also identified by multiplex PCR as described before. However, animals 9, 13 and, 14 showed negative results in this way. The reason for failure in this direct PCR typing might be likely due to the insufficient bacterial load in the tissue. In the revised version, we added the related information in section 2.2 and corrected the column title ‘PCR typing’ to ‘tissue direct PCR typing’ for S1 Table.

In addition, since the 17 serum samples collected from the 17 cattle bacterially identified to be infected by *M. bovis* through bacterial culture and genotyping which is considered as the gold standard method, we think the result difference between the two methods can reflect the differential sensitivity.

13. In section 3.3, I'd be interested to know the difference between goats and sheep as the data is now compounded together, whereas I believe sheep may be more often infected with *M. bovis* and goats with *M. caprae* (although I could be wrong?). Also, it would be interesting to see this compared to standard diagnostics for these animals? To see if the test performance of the MMEC-AG-HRP holds up.

Response 13: Thank you very much for this comment, and we are very sorry to cause this confusion.

You might be correct that sheep may be more often infected with *M. bovis*, whereas goats with *M. caprae*. Because the bacteriological evidence on sheep or goat infection by *M. bovis* or *M. caprae* is very limited in our study, currently we can’t conclude preference or susceptibility difference of sheep and goats to *M. bovis* or *M. caprae* infection. But based on the literature, both sheep (Sabrina Rodríguez, 2011; Ana Balseiro, 2017) and goats (Crawshaw T, 2008; R. Daniel, 2015) are susceptible to infect either *M. bovis* or *M. caprae*.

We are sorry to mix the results of sheep and goat results. Actually, the positive serum samples were mainly from sheep, therefore to avoid confusion, we deleted the results of goats in the revised version and added the test detailed results of sheep in S7 Table.

We agree it is very interesting to differentiate the susceptibility difference of sheep and goats to infect *M. bovis* or *M. caprae*. As mentioned above, we tried to isolate MTBC strains from 188 tissue samples of goats and sheep collected at some abattoir but failed to get any MTBC strains. Alternatively, the positive sera of the sheep were developed by immunization of PPD-B. Therefore, the current data can’t compare the difference of this assay to detect *M. bovis* or *M. caprae* infection. However, we will continue to investigate it in the future.

14. Figure 3 needs a lot more information: What samples do the different lanes in the gel represent? Where are the positive and negative controls and which lanes are they in? Which gene target(s) was/were amplified and what were the conditions for the PCR (DNA extraction method, PCR reagents used, cycling conditions etc).

Response 14: Thank you very much for this comment. We are very sorry to have neglected the related details about the multiplex PCR in Figure 3. The detailed information has been added in section 2.2 in the revised version as follows:

(1) Negative control was deionized water; *M. bovis* was isolated from the tuberculous nodule in the lung of a Pere David's deer; Positive control was *M. bovis* ATCC19210; *M. caprae* was isolated from the abscesses in the right submaxillary face of a roe deer. Lines: 1, *16S Rrna* (543 bp); 2, Rv0577 (786 bp); 3, IS*1561’* (943 bp); 4, Rv1510 (1033 bp); 5, Rv1970 (1116 bp); 6, Rv3877/8 (999 bp); 7, Rv3120 (404 bp). M, 2000 bp DNA ladder.

(2) Since the positive control represents *M. bovis* ATCC19210, while the negative control represents deionized water corresponding to each gene. We are sorry to cut the gel considering the large size of the original gel. In the revised version, we changed the picture to the original one with negative control and positive control.

(3) The seven target genes amplified by multiplex PCR are *16S Rrna,* Rv0577, IS*1561*, Rv1510, Rv1970, Rv3877/8, and Rv3120. The genome of the mycobacteria clone was extracted by boiling, and the subspecies were identified by multiplex PCR. Each PCR mixture was prepared with 25 μL of PCR Master (Tsingke Biotechnology Co., Ltd., China), 20 μL of water, 2.5 μL of dimethyl sulfoxide, 1 μL of each primer at 20 μM, and 0.5 μL of DNA. PCR amplifications were performed with an initial denaturation step of 5 min at 94°C followed by 35 cycles of 60 s at 94°C, 60 s at 60°C, and 90 s at 72°C, and ending with a final elongation step for 10 min at 72°C. PCR products were visualized by 1% agarose gel electrophoresis.

More specific comments:

Abstract:

Line 23: Could be changed to '..an ante-mortem technique..." and then delete "live" from the sentence.

Response: Thank you very much for this suggestion. This sentence has been rewritten as follows: Developing an ante-mortem detection technique for different species would be of significance in improving the surveillance employing a One Health strategy.

Line 24: Remove the words "effect based on" and change to "..employing a One Health.."

Response: Thank you very much for this suggestion. This sentence has been rewritten as follows: Developing an ante-mortem detection technique for different species would be of significance in improving the surveillance employing a One Health strategy.

Line 27: Brackets around MMEC are missing: (MMEC). Also, I think you mean "secondary antibody" or "detection antibody". the term "second antibody" appears in several places in the manuscript. Please correct everywhere.

Response: Thank you very much for this suggestion. Bracket has been added, and all the "second antibody" have been replaced with "secondary antibody".

Line 31: Change "Besides" to "Furthermore".

Response: Thank you very much for this suggestion. "Besides" has been changed to "Furthermore".

Introduction:

There do not appear to be any references for the first couple of sentences? Would be worth adding some here.

Response: Thank you very much for this suggestion. The reference has been added.

Line 38: Not sure I like the term "cross-infected", consider removing "cross-" and just saying "..infected by various.."

Response: Thank you very much for this suggestion. This sentence has been rewritten as follows: Tuberculosis (TB) is known as an important zoonotic infectious disease caused by various *Mycobacterium tuberculosis* complex (MTBC) members.

Line 50: Change "Besides" to "Additionally".

Response: Thank you very much for this suggestion. "Besides" has been changed to "Additionally".

Line 63: Change to "or are unsuitable" ("are" is missing from your sentence).

Response: Thank you very much for this suggestion. "are" has been added.

Line 70: Change "possibly used" to "possible candidate"

Response: Thank you very much for this suggestion. "possibly used" has been changed to "potential candidate".

Line 71: Change "etc" to "among others"

Response: Thank you very much for this suggestion. "etc" has been changed to "and others"

Line 76: Change "..addressing it is to develop antigen.." to "..addressing this is to develop an antigen..".

Response: Thank you very much for this suggestion. "it" has been changed to "this", and "an" has been added.

Line 78: Change "furthermore" to "however" and change "..antigen, might.." to "..antigen, it might.."

Response: Thank you very much for this suggestion. This sentence has been rewritten as follows: However, as mentioned above, by targeting a single antigen, it might not display sufficient sensitivity.

Line 80: Remove "by".

Response: Thank you very much for this suggestion. "by" has been removed.

Line 82: Remove "was".

Response: Thank you very much for this suggestion. "was" has been removed.

Line 86: Change "calibrated" to "optimized". This occurs in several places in the manuscript and should be revised everywhere.

Response: Thank you very much for this suggestion. The "calibrated" in different places have been replaced with "optimized".

Section 2.2

Line 94: Change "was" to "is".

Response: Thank you very much for this suggestion. "was" has been changed to "is".

Lines 109/110: Change "shorten" (which is grammatically incorrect) to "shortened" or "abbreviated".

Response: Thank you very much for this suggestion. "shorten" has been changed to "abbreviated".

Line 112: 72h should be written out as it is at the start of a sentence (so "Seventy-two hours).

Response: Thank you very much for this suggestion. "72h" has been changed to "Seventy-two hours".

Section 2.4

Line 144: Change "The 100" to "A volume of 100".

Response: Thank you very much for this suggestion. "The 100" has been changed to "A volume of 100".

Line 154: Blocking buffer is suddenly mentioned for the first time without being defined. Assume this is 1% fish gelatin in PBS?

Response: Thank you very much for this feedback. Blocking buffer was redefined as follows: then 200 μL blocking buffer (1% fish gelatin (Sigma, America) in PBS) was added and incubated for 1 h at 37°C.

Line 158: What is the concentration of Tween in PBS in the PBST for washing? In this section (and maybe others) you say "the plate is added with xxx" which is a bit odd. I would suggest to change this to "xxx was added to the plate". Please change this accordingly, throughout the manuscript.

Response: Thank you very much for this suggestion. The concentration of Tween in PBST (0.5% Tween-20 in PBS) was added into the text, and "the plate is added with xxx" has been changed to “xxx was added to the plate”.

Section 2.6

"respectively" is used a lot, but it is out of place. In the first sentence talking about the bovine samples, the use of "respectively" makes it seem as though you used 1:50 for the positive samples and 1:100 for the negative samples. Whereas I assume both dilutions were used for both positive and negative samples. Then for the other species it gets even more confusing as there are now 3 dilutions. I'd just remove "respectively" from all sentences. Also, why only these dilutions as opposed to a range? And to reiterate: please also show these data.

Response: Thank you very much for this suggestion. All the "respectively" have been deleted. Regarding dilution range, as explained in Question 2, we firstly used a range of dilutions for chessboard titration and determined the optimal dilution to be 1:100 when the P/N value was the largest and/or the OD value of the positive serum was slightly over 1. Then considering the difference in antibody response in different species, two or three dilutions around the optimal dilution were be used and the dilution with the highest sensitivity and specificity was selected for each species. In the revised submission, the additional data were added as the supplementary tables (Table S4 for bovines, S7 for sheep, S11 for cervids, S14 for monkeys, and S16 for humans).

Section 2.8

The heading "Application of MMEC/AG-iELISA to TB serological diagnosis" reads a bit funny. Maybe change it to something like "Application of MMEC/AG-iELISA for the serological diagnosis of TB".

Response: Thank you very much for this suggestion. The heading has been changed to "Application of MMEC/AG-iELISA for the serological diagnosis of TB".

Section 3.2

Line 223: "combinedly" is a very odd word. Revise.

Response: Thank you very much for this comment. "combinedly" has been deleted.

Line 249: "as a result" is not used appropriately here. Revise.

Response: Thank you very much for this comment. "as a result" has been changed to "of which".

Line 252: "no difference" should be "no significant difference".

Response: Thank you very much for this suggestion. "no difference" has been changed to "no significant difference".

Here "calibrated" is used a lot, but "optimized" is more fitting.

Response: Thank you very much for this suggestion. All the "calibrated" have been replaced with "optimized".

In table 2, in the headings of the columns, change "down" to "lower".

Response: Thank you very much for this suggestion. "Table 2" has been deleted.

Section 3.4

Line 294: "Detect" should be changed to "test" ("tests" should then maybe be changed to "assays").

Response: Thank you very much for this suggestion. This sentence has been rewritten as follows: Further, both assays were used to test the one roe deer serum infected with *M. caprae*.

Section 4

Line 315: I feel like it is a bit obvious that TB impedes the End-TB strategy? Make it clear that you make TB in animals versus TB in humans and the relationship between the two.

Response: Thank you very much for this suggestion. We are sorry to cause this confusion. The relationship between the two is re-written as follows: “Since TB is an important zoonotic disease, maintenance of MTBC infection in animals is a great barrier for achieving this goal of the End-TB strategy.”

Line 328: Change "were" to "is".

Response: Thank you very much for this suggestion. "were" has been replaced with "is".

Line 342: Remove "be".

Response: Thank you very much for this suggestion. "weren’t be" has been changed to "wouldn’t have been".

Lines 345 and 356: Change "Alternatively" to "As an alternative".

Response: Thank you very much for this suggestion. "Alternatively" has been changed to "As an alternative".

Line 348: This sentence is too long and complex. End the sentence after "*M. tb* infection." and then start the new sentence "Different...." (so remove "Although).

Response: Thank you very much for this suggestion. The correction has been made as suggested.

Line 364: "As a result" is not necessary here and a bit out of place.

Response: Thank you very much for this suggestion. "As a result" has been deleted.

Line 384: Change "Besides sheep and goats are susceptible." to "Besides sheep and goats, which are susceptible." or similar.

Response: Thank you very much for this suggestion. This sentence has been rewritten as follows: In addition to sheep and goats, which are susceptible hosts of *M. caprae* [42], cervids such as red deer are natural hosts and maintenance reservoirs of *M. caprae* [43].

Special thanks to you for your good comments.

Response to Reviewer 2 Comments

Reviewer #2: This was a very interesting paper which investigates the development of a multi-TB species ELISA using multiple antigens on a single plate. It will be a welcomed addition to an area where simple diagnostics are sorely lacking.

Author response: We greatly appreciate for the positive comments.

Major points

1.I am surprised that you only test a few dilutions for the titrations. Was there a reason for this? I would have expected to see more.

Response 1: Thank you very much for this comment.

We are very sorry to have neglected the related background about the serum dilution and completely agree with you that a range of serum dilutions should be used in a checkerboard titration to determine the optimal serum dilution. We actually followed this standard in this study. To determined optimum serum dilution, we performed checkerboard titration by using a range of serum dilutions of the positive bovine serum (the pooled 120 positive bovine antisera) and negative bovine serum (the pooled 242 negative bovine sera) since the amount of these bovine sera could meet demands of repetitive tests. As a result, the optimal dilution was determined to be 1:100 (the addition table named as S3 table was provided in the revised version). Considering the difference of serum titers among various species, in practical tests for each species, the two or three dilutions are selected around the 1:100. Then sensitivity and specificity were compared and the dilution with the highest sensitivity and specificity was finally determined for each species. We added the ROC curves for other serum dilutions in the tests of each species to better show the evidence for decision-making (please see Fig.2, 3, 5, and 6).

2.As a question, would the assay detect organisms such as *M. avium sub. Paratuberculosis* (Johnes disease)? This would be of interest too. And how does it cope with a co-infection?

Response 2: Thank you very much for this comment. In the specificity test (section 3.2), the cattle antiserum to *M. paratuberculosis* was tested by this MMEC/AG-iELISA and determined to be negative. Therefore, *M. paratuberculosis* infection will not affect the test result of MMEC/AG-iELISA.

3.I think stating that the assay will aid in disease control may be a bit over the top. Although they do slaughter based on the skin tests, it may be that this assay would supersede this, but maybe worth edging on the side of caution.

Response 3: Thank you very much for this comment. We agree with you and the description has been changed as follows: “Thus this method has a promising application in serological TB surveillance for multiple animal species thereby providing evidence for taking further action in TB control.”

Line 23- improving the surveillance effort based on a One Health strategy (reword)

Response: Thank you very much for this suggestion. This sentence has been rewritten as follows: Developing an ante-mortem detection technique for different species would be of significance in improving the surveillance employing a One Health strategy.

Line 25- please define MTBC in the abstract

Response: Thank you very much for this suggestion. This has been corrected.

Line 27- as the secondary antibody (reword)

Response: Thank you very much for this suggestion. This has been corrected.

Line 28- comma after then

Response: Thank you very much for this suggestion. This has been corrected.

Line 30- some values in here may be useful

Response: Thank you very much for this comment. As suggested, we added the values. This sentence has been rewritten as follows: After testing the known positive and negative sera, the receiver operating characteristic curves were constructed to decide the cut-off values. Then, the diagnostic sensitivity and specificity of MMEC/AG-iELISA were determined as 100.00% (95% CI: 96.90%, 100.00%) and 100.00% (95% CI: 98.44%, 100.00%) for *M. bovis* infection of cattle, 100.00% (95% CI: 95.00%, 100.00%) and 100.0% (95% CI: 96.80%, 100.00%) for *M. bovis* infection of sheep, 90.74% (95% CI: 80.09%, 95.98%) and 98.63% (95% CI: 95.14%, 99.76%) for *M. bovis* infection of cervids, 100.00% (95% CI: 15.81%, 100.00%) and 98.81% (95% CI: 93.54%, 99.97%) for *M. bovis* infection of monkeys, 100.00% (95% CI: 86.82%, 100.00%) and 94.85% (95% CI: 91.22%, 97.03%) for *M. tb* infection of humans.

Line 31- in addition may sound better than besides.

Response: Thank you very much for this suggestion. "besides" has been replaced with "Furthermore" as suggested by the reviewer 1 which is close to your kind suggestion “in addition”.

Line 38-40- could you link some of these short sentences to aid flow for the reader? Also *M. bovis* and *M. caprae* need to be in full on their first usage. I also feel that these may benefit from some references being inserted

Response: Thank you very much for this suggestion. We are awfully sorry for this confusion. As suggested, some information was added to aid flow for the reader. The full names *Mycobacterium bovis* (*M. bovis*), and *Mycobacterium caprae* (*M. caprae*) were added. In addition, the reference has been added. This sentence has been rewritten as follows: The main members of MTBC include *Mycobacterium tuberculosis* (*M. tb*), *Mycobacterium bovis* (*M. bovis*), and *Mycobacterium caprae* (*M. caprae*), which share a wide range of hosts but maintain different host preferences [1]. For example, human TB (hTB) is mainly caused by *M. tb*, but *M. bovis* of animal origin can infect humans and cause hTB either*.*

Line 43- this reads a bit awkwardly, please reword

Response: Thank you very much for this suggestion. This sentence has been rewritten as follows: Among the hTB cases, the proportions of cases caused by *M. bovis* infection varied in different countries and areas.

Line 49- delete the

Response: Thank you very much for this suggestion. "the" has been deleted.

Line 51- maybe susceptible rather than sensitive?

Response: Thank you very much for this suggestion. "sensitive" has been replaced with "susceptible".

Line 52-53- this reads a bit awkwardly, please reword

Response: Thank you very much for this suggestion. This sentence has been rewritten as follows: Therefore, OIE added the infection of bovids, cervids, goats, and New World camelids by *M. bovis*, *M. tb*, and *M. caprae* into the disease list of the Terrestrial Animal Health Code since 2019.

Line 61- such as the tuberculin skin … (reword)

Response: Thank you very much for this suggestion. "the" has been added.

Line 63- either have low sensitivities… (reword)

Response: Thank you very much for this suggestion. "have either low sensitivities" has been changed to "either have low sensitivities".

Line 63- or are unsuitable for the …. (reword)

Response: Thank you very much for this suggestion. "are" has been added.

Line 65- this isn’t completely true for animal TB. I also don’t believe that the *M. bovis* vaccine is used much- but I could be incorrect.

Response: Thank you very much for this suggestion and we agree with your points. This sentence has been rewritten as follows by deleting the last part about BCG: Antibody detection is an ideal choice to identify the risk of TB transmission in multiple species due to its advantages of simple, easy, rapid, and high-throughput screening.

Line 67- comma after screening

Response: Thank you very much for this suggestion. This has been corrected.

Line 67- is this *M. bovis*, or should it be *M. tb*?

Response: Thank you very much for this suggestion. The formal full name of BCG is *M. bovis* BCG. In addition, this sentence has been rewritten as follows by deleting the last part about BCG: Antibody detection is an ideal choice to identify the risk of TB transmission in multiple species due to its advantage of simple, easy, rapid, and high-throughput screening.

Line 68- low sensitivity may sound better than lowly?

Response: Thank you very much for this suggestion. "lowly sensitive" has been changed to "low sensitivity".

Line 70- the potential antigens may sound better?

Response: Thank you very much for this suggestion. It has been corrected.

Line 70- were these antigens used for all host species?

Response: Thank you very much for this comment. We are awfully sorry for this confusion. These antigens were not equally used in all host species, humans and bovines are more common. We listed some host species and added the references.

Line 74- limitation of the antibody test with a traditional … (reword)

Response: Thank you very much for this suggestion. This sentence has been rewritten as follows: Another limitation of the antibody test with a traditional ELISA lies in its species specificity of the secondary antibodies which varies with host species.

Line 76- develop an antigen … (reword)

Response: Thank you very much for this suggestion. "an" has been added.

Line 79- comma after test

Response: Thank you very much for this suggestion. This sentence has been rewritten as follows: However, as mentioned above, by targeting a single antigen, it might not display sufficient sensitivity.

Line 80- delete by

Response: Thank you very much for this suggestion. "by" has been deleted.

Line 81- overcome the Ig species ….(reword)

Response: Thank you very much for this suggestion. It has been corrected.

Line 82- aimed to develop a single universal ….(reword)

Response: Thank you very much for this suggestion. "one universal" has been changed to "a single universal".

Line 85- maybe worth saying A and G proteins of which bacteria

Response: Thank you very much for this comment. We have changed to *Staphylococcal* protein A and/or *Streptococcal* protein G.

Line 85- bind to Ig of various host species …. (reword)

Response: Thank you very much for this suggestion. "bind to Ig of various species of host" has been changed to "bind to Ig of various host species".

Line 94- is presented in table 1 (reword)

Response: Thank you very much for this suggestion. "was" has been changed to "is".

Line 94- how were the human positive and negative sera identified? Was this by PCR or an ELISA? Please state in here. This is also true for other species

Response: Thank you very much for this comment. We are very sorry to have neglected the related background details about the human serum production. Yes, the human TB patients were confirmed with *M. tb* infection and molecular techniques. We added the detail of typing here and other places too.

Line 94- comma after sera

Response: Thank you very much for this suggestion. This has been corrected.

Line 98- is seropositive and TB positive the same here?

Response: Thank you very much for this comment. It is the same here.

Line 99- in vitro in italics

Response: Thank you very much for this suggestion. This has been corrected.

Line 101- and were confirmed as ….(reword)

Response: Thank you very much for this suggestion. This has been corrected.

Line 103- confirmed by the comparative …(reword)

Response: Thank you very much for this suggestion. "the" has been added.

Line 103- in vitro in italics

Response: Thank you very much for this suggestion. This has been corrected.

Line 103- and with the above mentioned ELISA kits (reword)

Response: Thank you very much for this suggestion. This has been corrected.

Line 104- this is repetitive and could be removed

Response: Thank you very much for this suggestion. "and these sera were collected from TB-free dairy cows" has been removed.

Line 105- *B. abortus* in brackets isn’t needed and can be deleted

Response: Thank you very much for this suggestion. *B. abortus* in brackets has been deleted.

Line 109- maybe PPD isn’t the best phrasing- perhaps just say TB? Or explain what PPD is

Response: Thank you very much for this suggestion. We have explained that PPD is tuberculin purified protein derivative.

Line 114- positive sheep sera (reword)

Response: Thank you very much for this suggestion. This has been corrected.

Line 115- what’s PPD-B?

Response: Thank you very much for this comment. PPD-B is the abbreviation of bovine tuberculin purified protein derivative and has been redefined when it first appeared.

Line 117-121- I found this unclear, so please reword

Response: Thank you very much for this suggestion. This sentence has been rewritten as follows: Besides, one TB negative sheep was immunized with the mixture of 10 mL recombinant fusion protein MMEC (0.2 mg/mL) and 10 mL Freund's complete adjuvant (Sigma, America), and boosted with the mixture of the same amount of MMEC and Freund's incomplete adjuvant (Sigma, America) 15 days later, the positive sera were collected one week later and used as a positive control. Meanwhile, the serum sample collected from the sheep before immunization with MMEC was used as a negative control.

Line 122- remove were

Response: Thank you very much for this suggestion. "were" has been deleted.

Line 123- one positive serum sample to … (reword)

Response: Thank you very much for this suggestion. "sample" has been added.

Line 128- remove both ‘were’

Response: Thank you very much for this suggestion. Both "were" have been deleted.

Table 1- could this be separated more for clarity?

Response: Thank you very much for this suggestion. To make Table 1 easier to be understood, we added related information on the background of serum collection in the revised version. To give the readers a whole picture of the serum samples from different hosts, we think it might be better to keep it as the present status. Therefore, would you please allow us to keep it?

Please ensure that *in vitro* is in italics in Table 1

Response: Thank you very much for this suggestion. This has been corrected.

Also include a dash in table one where there are blank cells

Response: Thank you very much for this suggestion. Dash has been added.

Line 136- is there a reference for that?

Response: Thank you very much for this suggestion. Reference has been added.

Line 138-139- please include manufacturers in here

Response: Thank you very much for this suggestion. The manufacturer has been added.

Line 144- 100 μL of 20 mg … (reword)

Response: Thank you very much for this suggestion. "of" has been added.

Line 144- please have NaIO4 in full

Response: Thank you very much for this suggestion. This has been corrected.

Line 147- 1 mL of 2mg …(reword)

Response: Thank you very much for this suggestion. "of" has been added.

Line 148- please have NaBH4 in full

Response: Thank you very much for this suggestion. The full name has been added.

Line 150- maybe incubated or mixed rather than added

Response: Thank you very much for this suggestion. "added" has been changed to "mixed".

Line 154- maybe incubated or mixed rather than added

Response: Thank you very much for this suggestion. This “added” has been replaced.

Line 155- immune with MMEC- maybe reword this as it seems unclear

Response: Thank you very much for this suggestion. "immune with MMEC" has been deleted.

Line 157- then the plate was incubated with? (maybe reword)

Response: Thank you very much for this suggestion. This sentence has been rewritten as follows: Then the plate was incubated with 100 μL of two-fold serially diluted AG-HRP (diluted with blocking buffer) for 30 min at 37°C.

And 159- and 160

Response: Thank you very much for this suggestion. These sentences have been rewritten as suggested.

Line 161- is this 1M or 1 N?

Response: Thank you very much for this comment. According to the specification of SureBlue TMB microwell substrate, it is 1 N.

Line 171- delete as

Response: Thank you very much for this suggestion. "as" has been deleted.

Line 171- diluted in what?

Response: Thank you very much for this suggestion. The positive and negative sera were diluted with blocking buffer. This sentence has been rewritten as follows: The positive and negative sera of each species described above were diluted with blocking buffer as follows.

Line 182- deer sera was determined …(reword)

Response: Thank you very much for this suggestion. "sera" has been added.

Line 183-184- Please reword as this reads awkwardly

Response: Thank you very much for this suggestion. This sentence has been rewritten as follows: In addition, the cut-off value of monkey sera was alternatively defined as the mean S/P value of the negative sera plus 3 standard deviations (SD) with 99% confidence due to the insufficient positive sera [24].

Line 196- in addition may sound better than besides

Response: Thank you very much for this suggestion. "besides" has been changed to "in addition".

Line 204 and 207- please include the manufacturer of these stats packages

Response: Thank you very much for this suggestion. The manufacturer has been added.

Line 211- *E. coli* (include space)

Response: Thank you very much for this suggestion. This has been corrected.

Line 211- maybe purified to a stocking concentration?

Response: Thank you very much for this suggestion. "with" has been changed to "to".

Line 213- please reword as this doesn’t make sense

Response: Thank you very much for this suggestion. This sentence has been rewritten as follows: Meanwhile, the titer of AG-HRP was determined as 1×2^21^ with a stocking concentration of 4.02 mg/mL.

Figure 1- is that 75KDa? It looks larger than that but may be the image?

Response: Thank you very much for this comment. The recombinant plasmid was previously constructed in our lab, encoding the fusion protein MMEC which includes *mpb70*, *mpb83*, *cfp10*, and *esat6* gene, and the linker GSGGGGSGGGGSGS between each two genes. We confirmed the plasmid to be correct by digestion with *EcoRI* and *HindⅢ* and analysis on the coding sequence of MMEC by sequencing. Therefore, we think it might be the concentration of SDS-PAGE causing this difference. Then we re-ran the 10% SDS-PAGE and got the expected size of the band.

Line 223- combinedly confirmed sounds strange- please reword

Response: Thank you very much for this suggestion. "combinedly" has been deleted.

Line 224- please put *in vitro* in brackets.

Response: Thank you very much for this suggestion. A bracket has been added.

Table 2- is there any reason why cervids detection limits are lower?

Response: Thank you very much for this comment. We didn’t know the exact reason. Compared to the development of other positive serum samples, it may be related to the limited methods to define the positive serum samples of cervids which included MPB83 sandwich ELISA and PPD-ELISA. The sensitivity of both methods could be relatively low.

Line 255- negative sheep sera (reword)

Response: Thank you very much for this suggestion. This has been corrected.

Line 256- positive sheep sera (reword)

Response: Thank you very much for this suggestion. This has been corrected.

Line 267- why only one sample used here? I would like to see more used for confirmation

Response: Thank you very much for this comment. This assay was used to only compare analytical sensitivity (the lowest detection dilution, or limit of detection). Since the positive serum needs to be two-fold serially diluted which represents a lot of work, we used one positive serum sample for each species.

Line 282- this reads awkwardly, please reword

Response: Thank you very much for this suggestion. This sentence has been rewritten as follows: The cervid TB diagnosis was optimized to be as follows.

Line 287- I feel that figure 3 needs more information what is it showing, what is in each lane, controls? Sizes, targets etc as well as methodology for production

Response: Thank you very much for this comment. We are very sorry to have neglected the related background details about Figure 3. We have added all the suggested information in the revised version as follows:

(1) Different lanes represent different target gene amplified by multiplex PCR. Lanes: 1, *16S Rrna* (543 bp); 2, Rv0577 (786 bp); 3, IS*1561’* (943 bp); 4, Rv1510 (1033 bp); 5, Rv1970 (1116 bp); 6, Rv3877/8 (999 bp); 7, Rv3120 (404 bp); M represent the 2000 bp DNA ladders.

(2) The positive control represents *M. bovis* reference strain, while the negative control represents deionized water. We are sorry to cut the gel considering the large size of the original gel. In the revised version, we changed the picture to the original one with negative control and positive control.

(3) Target genes amplified by multiplex PCR are *16S Rrna,* Rv0577, IS*1561*, Rv1510, Rv1970, Rv3877/8, and Rv3120. The genome of the mycobacteria clone was extracted by boiling, and the subspecies were identified by multiplex PCR. Each PCR mixture was prepared with 25 μL of PCR Master (Tsingke Biotechnology Co., Ltd., China), 20 μL of water, 2.5 μL of dimethyl sulfoxide, 1 μL of each primer at 20 μM, and 0.5 μL of DNA. PCR amplifications were performed with an initial denaturation step of 5 min at 94°C followed by 35 cycles of 60 s at 94°C, 60 s at 60°C, and 90 s at 72°C, and ending with a final elongation step for 10 min at 72°C. PCR products were visualized by 1% agarose gel electrophoresis. In the revised version, we added the related information in section 2.2 as follows: The collected samples were homogenized and decontaminated with 4% sodium hydroxide. After neutralization and centrifugation, the sediment is inoculated on 7H11 medium (Becton Dickinson, Franklin Lakes, NJ) for a minimum of 8 weeks at 37°C with CO2 for MTBC culture. When growth is visible, the genome of the clone was extracted by boiling. Then, the subspecies of the MTBC were identified by multiplex PCR targeting *16S Rrna*, Rv0577, IS*1561’*, Rv1510, Rv1970, Rv3877/8, and Rv3120 gene as described before [27]. Briefly, each PCR mixture was prepared with 25 μL of PCR Master (Tsingke Biotechnology Co., Ltd., China), 20 μL of water, 2.5 μL of dimethyl sulfoxide, 1 μL of each primer at 20 μM, and 0.5 μL of DNA. PCR amplifications were performed with an initial denaturation step of 5 min at 94°C followed by 35 cycles of 60 s at 94°C, 60 s at 60°C, and 90 s at 72°C, and ending with a final elongation step for 10 min at 72°C. PCR products were visualized by 1% agarose gel electrophoresis.

Line 313- why no dilution series in here?

Response: Thank you very much for this comment. We are very sorry to have neglected the related background about the serum dilution and completely agree with you that a range of serum dilutions should be used in a checkerboard titration to determine the optimal serum dilution. We actually followed this standard in this study. To determined optimum serum dilution, we performed checkerboard titration by using a range of serum dilutions of the bovine positive serum (the pooled 120 bovine positive antisera) and bovine negative serum (the pooled 242 bovine negative sera) since the amount of these bovine sera could meet demands of repetitive tests. As a result, the optimal dilution was determined to be 1:100 (the addition table named as S3 table was provided in the revised version). Considering the difference of serum titers among various species, in practical tests for each species, the two or three dilutions are selected around the 1:100. Then sensitivity and specificity were compared and the dilution with the highest sensitivity and specificity was finally determined for each species. We added the ROC curves for other serum dilutions in the tests of each species to better show the evidence for decision-making (please see Fig.2, 3, 5, and 6).

Line 318- of a One Health Strategy (reword)

Response: Thank you very much for this suggestion. This has been corrected.

Line 322- with a known antibody …(reword)

Response: Thank you very much for this suggestion. "a" has been added.

Line 327- infected with *M. bovis* (reword)

Response: Thank you very much for this suggestion. "by" has been changed to "with".

Line 334- sensitivities of the IDEXX …(reword)

Response: Thank you very much for this suggestion. "the" has been added.

Line 340- space between M and bovis

Response: Thank you very much for this suggestion. This has been corrected.

Line 342- would not have (replace weren’t be)

Response: Thank you very much for this suggestion. "weren’t be" has been changed to "wouldn’t have".

Line 349- will this not depend on the patient’s disease status?

Response: Thank you again for this smart comment. Yes, we agree with you it depends on the patient’s disease status. We represent it with “disease stages and severity degrees”.

Line 354- short lifetime- sheep or the disease?

Response: Thank you very much for this comment. It means sheep and we have corrected it in the revised version.

Line 355- close contact or relationship to humans maybe?

Response: Thank you very much for this comment. We changed the expression as suggested.

Line 355- serum samples from live …(reword)

Response: Thank you very much for this suggestion. "of" has been changed to "from".

Line 361- of the INGEZIM kit …(reword)

Response: Thank you very much for this suggestion. "the" has been added.

Line 361- maybe the most probably reason? Or most likely reason?

Response: Thank you very much for this suggestion. "possible" has been changed to "likely".

Line 362- of the INGEZIM kit …(reword)

Response: Thank you very much for this suggestion. "the" has been added.

Line 366- with seasonal variation ….(reword)

Response: Thank you very much for this suggestion. This has been corrected.

Line 370- delete the

Response: Thank you very much for this suggestion. "the" has been deleted.

Line 372- maybe ‘wildlife can be very aggressive and are difficult to capture and handle, and this test?

Response: Thank you very much for this comment. We changed the description as suggested.

Line 375- in vitro in italics

Response: Thank you very much for this suggestion. This has been corrected.

Line 378- TB testing in the …(reword)

Response: Thank you very much for this comment. This sentence has been rewritten as suggested.

Line 379- is monkey TB rarer than the other types?

Response: Thank you very much for this critical comment. To our knowledge, wild monkeys might have a high prevalence of TB. However, we checked the monkeys raised as the experimental animals. These monkeys have experienced several rounds of quarantine and testing. Therefore, the prevalence in these monkeys is low.

Line 381- comma after both samples

Response: Thank you very much for this suggestion. Comma has been added.

Line 383- this doesn’t make sense- *M. caprae* infected TB? Please reword

Response: Thank you very much for this comment. This sentence has been rewritten as follows: Although *M. caprae*-caused TB has been added to the OIE disease list.

Line 384- although may sound better than besides?

Response: Thank you very much for this comment. This sentence has been rewritten as suggested.

Line 392- space between *M. bovis* and *M. tb*

Response: Thank you very much for this suggestion. This has been corrected.

Line 393- of a One Health strategy (reword)

Response: Thank you very much for this suggestion. "a" has been added.

Special thanks to you for your good comments.
